# Antidiabetic effect of fucoxanthin extracted from *Sargassum* *angustifolium* on streptozotocin‐nicotinamide‐induced type 2 diabetic mice

**DOI:** 10.1002/fsn3.2301

**Published:** 2021-05-06

**Authors:** Najme Oliyaei, Marzieh Moosavi‐Nasab, Ali Mohammad Tamaddon, Nader Tanideh

**Affiliations:** ^1^ Seafood Processing Research Group School of Agriculture Shiraz University Shiraz Iran; ^2^ Department of Food Science and Technology School of Agriculture Shiraz University Shiraz Iran; ^3^ Center for Nanotechnology in Drug Delivery School of Pharmacy Shiraz University of Medical Science Shiraz Iran; ^4^ Stem Cells Technology Research Center Department of Pharmacology School of Medicin Shiraz University of Medical Sciences Shiraz Iran

**Keywords:** antidiabetic and antiobesity effect, fucoxanthin, mice model, type 2 diabetes mellitus

## Abstract

This work aimed to study the antidiabetic effect of encapsulated fucoxanthin with porous starch (PS) in streptozotocin and nicotinamide‐induced type 2 diabetic mice. Fucoxanthin was extracted and purified from *Sargassum angustifolium* and encapsulated in porous starch (PS). Diabetic mice groups were gavaged daily with fucoxanthin (400 mg/kg), either free or encapsulated into PS, and metformin (50 mg/kg) for three weeks. The results exhibited that the fucoxanthin and fucoxanthin‐loaded PS markedly prevented the weight gain in treated groups (*p* < .05). Moreover, both free and encapsulated fucoxanthin could decrease the fasting blood glucose and increase the plasma insulin level similar to metformin (*p* < .05). In addition, total cholesterol, triglyceride, and low‐density lipoprotein were lower in the treated groups. These results confirm antiobesity effect of fucoxanthin by regulating lipid profile parameters. Moreover, the histopathology evaluation of pancreatic tissue in diabetic mice exhibited that oral administration of metformin and fucoxanthin caused regeneration of pancreatic beta cells. This study revealed the healthy effect of seaweed pigment as a suitable bioactive compound which can be used in functional foods for natural diabetes therapy.

## Graphical abstract

This study revealed the healthy effect of seaweed pigment, fucoxanthin, as suitable bioactive compound which can be used in functional foods for natural therapy. This study confirmed the antidiabetic activity of fucoxanthin in STZ‐induced diabetic mice model.

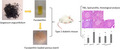

## 1 INTRODUCTION

Diabetes mellitus (DM) is a chronic metabolic disorder, characterized by hyperglycemia and glucose, protein, and fat metabolism disturbances which causes failure in insulin production, insulin action, or both (Farzaei et al., 2017). Type 2 diabetes is caused by β cell dysfunction and insulin resistance and is mostly associated with obesity. Thus, the treatments aiming to control obesity are considered in the therapy of type 2 diabetes (Q. Wang et al., 2019). Diabetic patients suffer from various disorders. Hence, their blood glucose level should be reduced and maintained in the normal range by different approaches, such as diet, medications, and exercise (Zaharudin et al., 2019).

In recent years, marine algae in particular brown seaweeds have been documented to possess health beneficial effect because of wide range of bioactive components such as catechins, phlorotannins, flavonoids, flavonols, and flavonol glycosides (Savaghebi et al., 2020) with antioxidant, anti‐inflammatory, antiobesity, and anticarcinogenic activities (S.‐y. Kang et al., 2018; Liu et al., 2020). Therefore, the seaweed industry has attracted much attention in development of functional materials for food, cosmeceutical, nutraceutical, and pharmaceutical industries (Liu et al., 2020). Several investigations reported that the supplementation of seaweeds or seaweeds extracts such as *Laminaria* *japonica* and *Hizikia fusiforme* (S.‐y. Kang et al., 2018), *Sargassum oligocystum* (Akbarzadeh et al., 2018), *Sargassum wightii* and *Ulva fasciata* (Mohapatra et al., 2016), *Gelidium amansii* (Yang et al., 2015) and *Ascophyllum nodosum* (Lordan et al., 2013) *Sargassum polycystum* (Motshakeri et al., 2013) exhibited antidiabetic activity by various pathways.

Among seaweed constituents, fucoxanthin is the most abundant carotenoid in brown macroalgae and has been considered because of its unique structure. Fucoxanthin metabolized to amarouciaxanthin A in adipose tissue and fucoxanthinol in other tissues (Miyashita et al., 2010). Moreover, fucoxanthin is safe under experimental condition in mice, and oral administration of fucoxanthin revealed no toxicity and mutagenicity (Martin, 2015).

In current years, several research has been claimed a possible biomedical potential and variety of different biological properties of fucoxanthin including antioxidative, anti‐inflammatory, anticancer, antiobesity, antidiabetic, antiangiogenic, and antimalarial attributes. Thus, this carotenoid exerts protective effect on different organs (Peng et al., 2011).

Among the extensive studies about its therapeutic activities, fucoxanthin has shown promising antidiabetic effect. A number of studies indicated that fucoxanthin exhibited the strong antiobesity and antidiabetic effects on diet‐induced obesity KK‐A^y^, C57BL/6N, C57BL/6J mice (Maeda et al., 2007, 2009; Woo et al., 2010). It has been suggested that the antidiabetic activity of fucoxanthin is associated with regulation of adipocytokine secretion, such as tumor necrosis factor‐α (TNF‐α) and interleukin 6 (IL‐6) and also modulation of monocyte chemoattractant protein‐1 (MCP‐1) mRNA expression in white adipose tissue (WAT), leading to prevention of hyperglycemia in a type 2 diabetes (Hosokawa et al., 2010; Maeda et al., 2009). In addition, fucoxanthin could decrease insulin resistance by increasing the hepatic glucokinase/glucose‐6‐phospahtase ratio and glycogen content (Park et al., 2011) and also regulating glucose transported 4 (GLUT4) mRNA expression in skeletal muscle tissues (Maeda et al., 2009).

Fucoxanthin is susceptible to light, oxygen, pH, and thermal degradation because of its multiple conjugated double bonds that could be problematic for long‐term storage (Quan et al., 2013). The evidence collected from other studies has confirmed the necessity of fucoxanthin encapsulation (Huang et al., 2017; Ravi et al., 2015; X. Wang et al., 2017). Encapsulation approaches protect the bioactive compounds from decomposition and interactions with other food components and improve their bioavailability and accurate release in food systems (Savaghebi et al., 2020). Porous starch (PS) is suggested as a new type of carrier for encapsulation of sensitive and poorly water‐soluble drugs (Wu et al., 2011). Our previous studies showed that the PS (Najme Oliyaei et al., 2020a) or modified PS with maltodextrin and gum Arabic (Oliyaei et al., 2020b) had appropriate encapsulation efficiency and in vitro release. Thus, in the present study, we aimed to assess the in vivo antidiabetic activity of fucoxanthin isolated from *Sargassum angustifolium*. Fucoxanthin was orally administered either as free or PS encapsulated powder to type 2 diabetic mice induced by streptozotocin (STZ) and nicotinamide (NA). Then, biochemical, histological, and morphometric alterations of pancreatic islets were evaluated. Besides, we conducted initial experiments to determine the effect of PS encapsulated fucoxanthin on reduction of the blood glucose in type 2 diabetic mice model.

## 2 MATERIALS AND METHODS

### 2.1 Materials

S. *angustifolium* was supplied by Algae Resource Development Technology Company (Shiraz, Iran). Standard fucoxanthin (>98%) and silica gel were purchased from J&K Scientific Ltd (China) and Nanochemia Company (Tehran. Iran), respectively. Ethanol (96% v/v), acetone, and n‐hexane were obtained from Dr. Mojallali Chemical Laboratories (Tehran, Iran). Streptozotocin (STZ) was purchased from Sigma‐Aldrich. Nicotinamide (NA), HPLC grade methanol, and acetonitrile were supplied from Merck Co.

### 2.2 Extraction and purification of fucoxanthin

Extraction of fucoxanthin was carried out according to S. M. Kim et al., (2012) with a slight modification. The powdered *S*. *angustifolium* was extracted three times (1:20 w/v) with 90% ethanol and filtered. Then, the concentrated extract was purified by column chromatography with silica gel (particle size 100–200 mesh) as normal stationary phase and n‐hexane‐acetone (6:4; v/v) as mobile phase. Finally, residual fucoxanthin was eluted by acetone. Purification analysis of fucoxanthin was performed with analytical HPLC system (KNAUER, Germany) equipped with UV/Vis detector 2,600 and C18 column (sphere‐image, ODS‐2, 300 x4 mm; 5 μm) and methanol/acetonitrile (50:50 v/v) as the mobile phase which was eluted at a 0.6 ml/min flow rate. Fucoxanthin was detected at 450 nm. and the results were compared with standard fucoxanthin (Norra et al., 2017). The recovered fucoxanthin exhibited the purity about 54% based on HPLC analysis of the area under the fucoxanthin curve.

### 2.3 Preparation of PS

PS was prepared according to the previous study (N Oliyaei et al., 2019); the mixture of corn starch and water (5% w/v) was heated at 90°C for 0.5 hr and then chilled at 5°C for 48 hr. The cylinder cut gels (about 1 × 1 cm) were frozen and finally subjected to solvent exchange by ethanol (100%). Immersion of gels in ethanol was carried out three times, for about 1h each time. The products were freeze‐dried.

### 2.4 Encapsulation of fucoxanthin

Fucoxanthin encapsulation was carried out according to Wu et al., (2011) with slight modification. The dried PS was mixed with fucoxanthin solution and then stirred overnight in the dark condition which allowed to reach equilibrium. Finally, samples were dried at a vacuum dryer.

### 2.5 Induction of diabetes

Six‐ to eight‐week‐old BALB/C mice (male, *n* = 30, w = 21.50 ± 3 g) were housed in a facility on a 12 hr light‐dark cycle with free access to food and water and controlled room temperature. Then, type 2 diabetes was induced by intraperitoneal injection of STZ (65 mg/kg body weight) freshly dissolved in a citrate buffer (0.1 M, pH 4.5) on three consecutive days, 15 min after a single dose of NA 110 mg/kg body weight in 0.1 ml normal saline on the first day. Diabetes was assured after a week by the daily measurement of blood glucose levels by glucose meter (Infopia EasyGluco AutoCoding Blood Glucose Meter, Korea), and mice with a blood glucose concentration above 180 mg/ml were considered to be type 2 diabetic (Lee et al., 2010).

### 2.6 Experimental design

To assess the antidiabetic activity of fucoxanthin, the mice were randomly allocated into five groups of six animals: Group 1: Normal control, Group 2: diabetic mice as negative control, Group 3: diabetic mice treated with metformin as positive control (received 50 mg/kg in 0.3 ml normal saline), Group 4: diabetic mice treated with fucoxanthin at dose of 400 mg/kg BW, Group 5: diabetic mice treated with PS encapsulated fucoxanthin at dose of 400 mg/kg BW, received daily gavage for 3 weeks after induction of type 2 diabetes.

### 2.7 Biochemical assessment

The body weight of animals was monitored on the final day of the experimental period. Also, after 24 hr of the last dose administered, blood samples of fasted mice were collected by heart puncture under acepromazine (80 mg/kg) and ketamine (100 mg/kg). Blood samples were centrifuged at 1512 g for 10 min for separation of sera. Each serum sample was stored at −80°C until further analysis. Serum was used to perform biochemical analysis, fasting blood glucose (FBG), fasting insulin, triglycerides (TG), total cholesterol (TC), high‐density lipid (HDL), and low‐density lipid (LDL) using commercially available kits.

### 2.8 Histopathology of pancreatic tissue

The pancreas tissue of each mouse was removed and fixed in buffer solution of 10% formalin. Fixed tissues were processed using paraffin embedding and sectioned at 4 μm. Sections of pancreas were stained with hematoxylin and eosin (H&E). Stained areas were observed using an optical microscope with a magnifying power of ×20.

### 2.9 Statistical analysis

The values were evaluated by one‐way ANOVA analysis followed by Duncan's multiple range tests. All the results were expressed as mean ± *SD* and analyzed using SAS software (SAS Institute). *p* values <.05 were considered statistically significant.

## 3 RESULTS AND DISCUSSION

### 3.1 Effect of fucoxanthin on body weight

**FIGURE 1:**
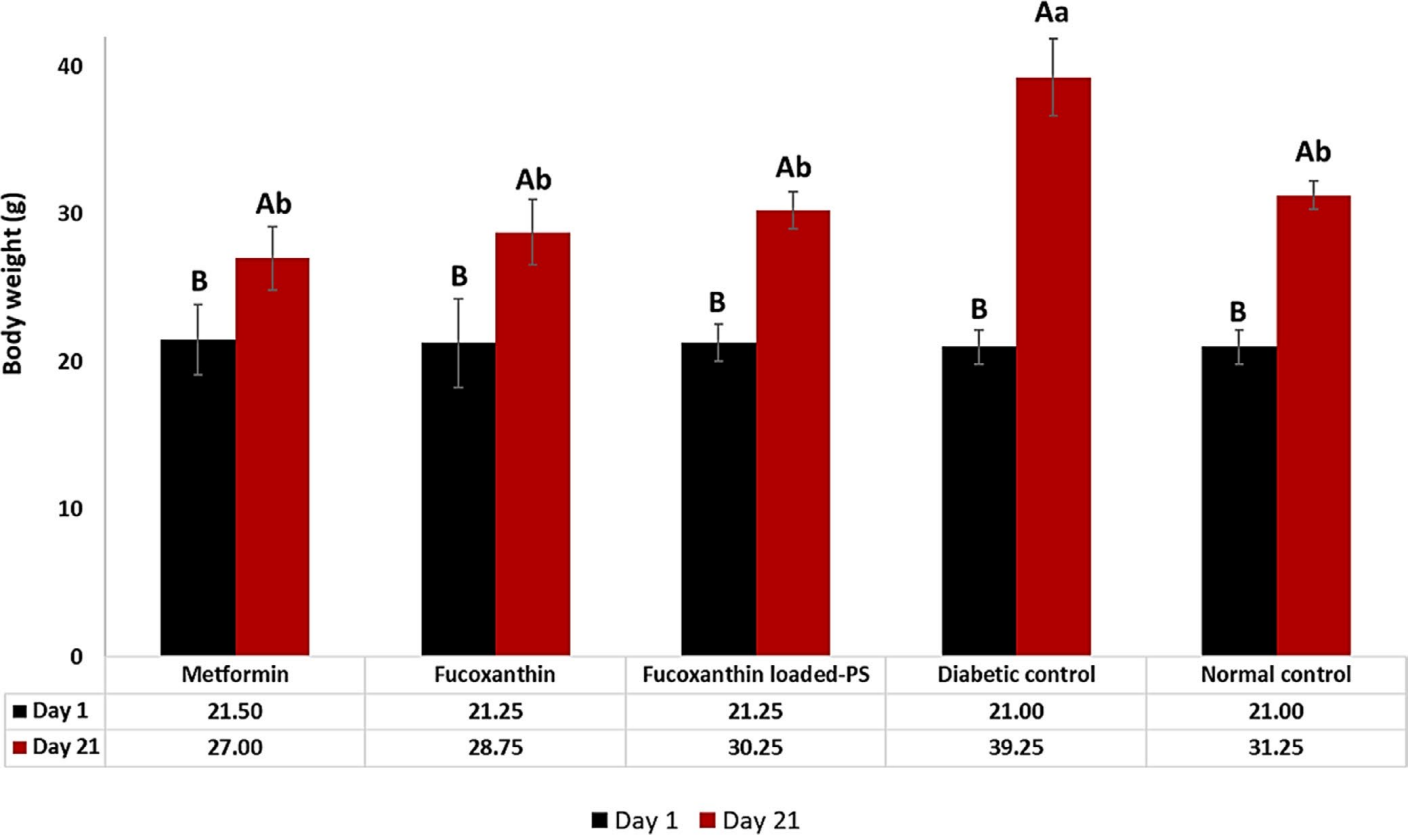
Body weight of different treatment groups; PS: porous starch. Data are expressed as mean ± *SD* (*n* = 3). Different capital letters in each groups and small letters between different groups indicate significant differences (*p* < .05)

The effect of fucoxanthin on the body weight of normal and diabetic mice is presented in Figure 1. Diabetic mice had greater body weight gain than normal group after 3 weeks (Figure 1, *p* < .05). Interestingly, no significant differences were observed between normal control and treatment groups. Among the treatments, fucoxanthin depicted a bit lower level of weight gain. A similar effect of fucoxanthin on body weight was reported by Iwasaki et al., (2012) in obese/diabetes KK‐*A^y^* mice model. Indeed, fucoxanthin could alter the leptin level and control body weight through the regulation of energy expenditure (Park et al., 2011).

### 3.2 Effect of fucoxanthin on FBS and plasma insulin levels

**FIGURE 2:**
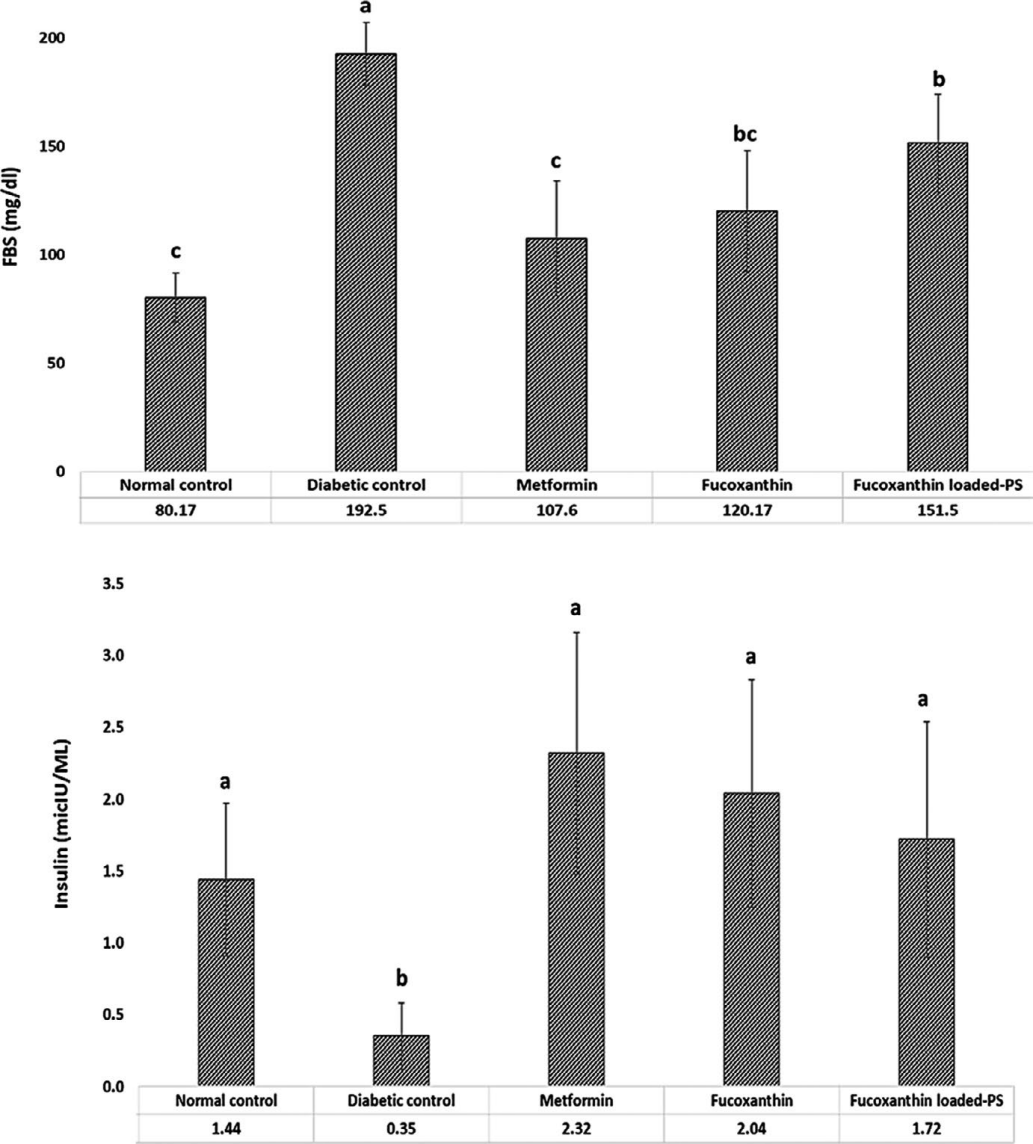
Fasting blood glucose (FBS) and insulin level of different treatment groups; PS: porous starch. Data are expressed as mean ± *SD* (*n* = 3). Different letters indicate significant differences (*p* < .05)

Effect of fucoxanthin on FBS and plasma insulin is shown in Figure 2. Fasting blood glucose level was increased significantly in diabetic group compared with normal group after three weeks (*p* < .05). All treated groups showed statistically significant reduction in FBS compared with the diabetic group (*p* < .05); however, there was no significant difference between metformin and fucoxanthin treated groups. Furthermore, the plasma insulin level of treated mice was higher than diabetic control mice. Fucoxanthin administration, either free or PS encapsulated, increased the plasma insulin level similar to metformin (*p* > .05). Similar results have been attained by dietary fucoxanthin in KK‐A^y^ mice (Maeda et al., 2007). Several mechanisms of action explaining the fucoxanthin antidiabetic effects have been suggested. There are two main mechanisms of antidiabetic effect of fucoxanthin: downregulation of adipokines RNA expression such as TNF‐α and MCP‐1 and overexpression of GLUT‐4 (Maeda et al., 2009). Hosokawa et al., (2010) reported that the weight gain and blood glucose level decreased after administration of 0.2% fucoxanthin in diabetic/obese KK‐A^y^ mice. Moreover, dietary fucoxanthin caused reduction of MCP‐1, TNF‐α, IL‐6, and plasminogen activator inhibitor‐1 (PAI‐1) expression in perigonadal WAT. In addition, MCP‐1 is known to mediate macrophage migration which attributed to insulin resistance and type 2 diabetes. Moreover, fucoxanthin conversion to fucoxanthinol by lipase and esterase secreted from pancreas or intestine (H. Zhang et al., 2015) can suppress the MCP‐1 production (Hosokawa et al., 2010). Also, fucoxanthinol can directly stop macrophage infiltration in WAT by reducing the mRNA expression level of the pro‐inflammatory adipocytokines. Besides, fucoxanthinol indirectly inhibits the macrophages infiltration by reducing the adipocytes production, because adipocytes release saturated fatty acids, such as palmitic acid which activate macrophages infiltration into WAT (Hosokawa et al., 2010). Other factors such as the presence of fish oil can influence on the fucoxanthin function as reported by Maeda et al., (2007). They gained a better profile of blood glucose and plasma insulin in KK‐A^y^ mice fed with 0.1% fucoxanthin in fish oil. Also, the upregulation of insulin receptor substarte‐1 (IRS‐1) was reported in *db/db* mice treated with fucoxanthin which is related to the reduction of blood glucose and insulin resistance (Lin et al., 2017).

Another possible mechanism of fucoxanthin in antidiabetic effect may be due to inhibition or decreased activity of α‐glycosidase activity that converts carbohydrates into glucose. Therefore, increased blood glucose level is prevented by slowing down the carbohydrate absorption in the small intestine (Zaharudin et al., 2019). Zaharudin et al., (2019) reported a strong α‐glucosidase inhibitory activity of fucoxanthin with a lower IC_50_ value than the specific inhibitor acarbose. They also reported that administration of acetone extract of brown seaweeds such as *Undaria pinnatifida* and *Laminaria digitate* effectively inhibited α‐glucosidase. However, *U*. *pinnatifida* extract had significantly inhibitory activity (>70%). Moreover, they suggested that the brown algae extract had better inhibitory effects compared with red algae (Zaharudin et al., 2019).

Moreover, α‐amylase inhibitors are considered for hyperglycemia treatment because help to slow carbohydrate digestion and prevent the entry of glucose into the circulation (Gong et al., 2020). In the present study, our fucoxanthin enriched extract contained polyphenolic compounds, such as phlorotannins which can act as inhibitors of α‐amylase and α‐glucosidase (Parada et al., 2019). However, seaweed extracts have different inhibitory effect on variety of enzymes, which is depending on the seaweed's polyphenol profile (Lordan et al., 2013). Furthermore, phenolic compounds play an important role on several sodium‐dependent glucose transporters (GLUT) that may limit glucose absorption in the intestine (Gauer et al., 2018). Stimulation of insulin secretion, hepatic glucose output reduction, enhanced insulin‐dependent glucose uptake, activation of 5′ adenosine monophosphate‐activated protein kinase (AMPK) are also reported which can be due to their antioxidant and anti‐inflammatory activity (Y. Kim et al., 2016). Similarly, the effect of other seaweeds extracts such as *U*. *prolifera* (Song et al., 2018), *Capsosiphone fulvescens*, *Hizikia fusiforme,* and *U*. *pinnatifida* (Tong et al., 2015) on improving the glucose level and insulin sensitivity were reported.

### 3.3 Lipid profile

**FIGURE 3:**
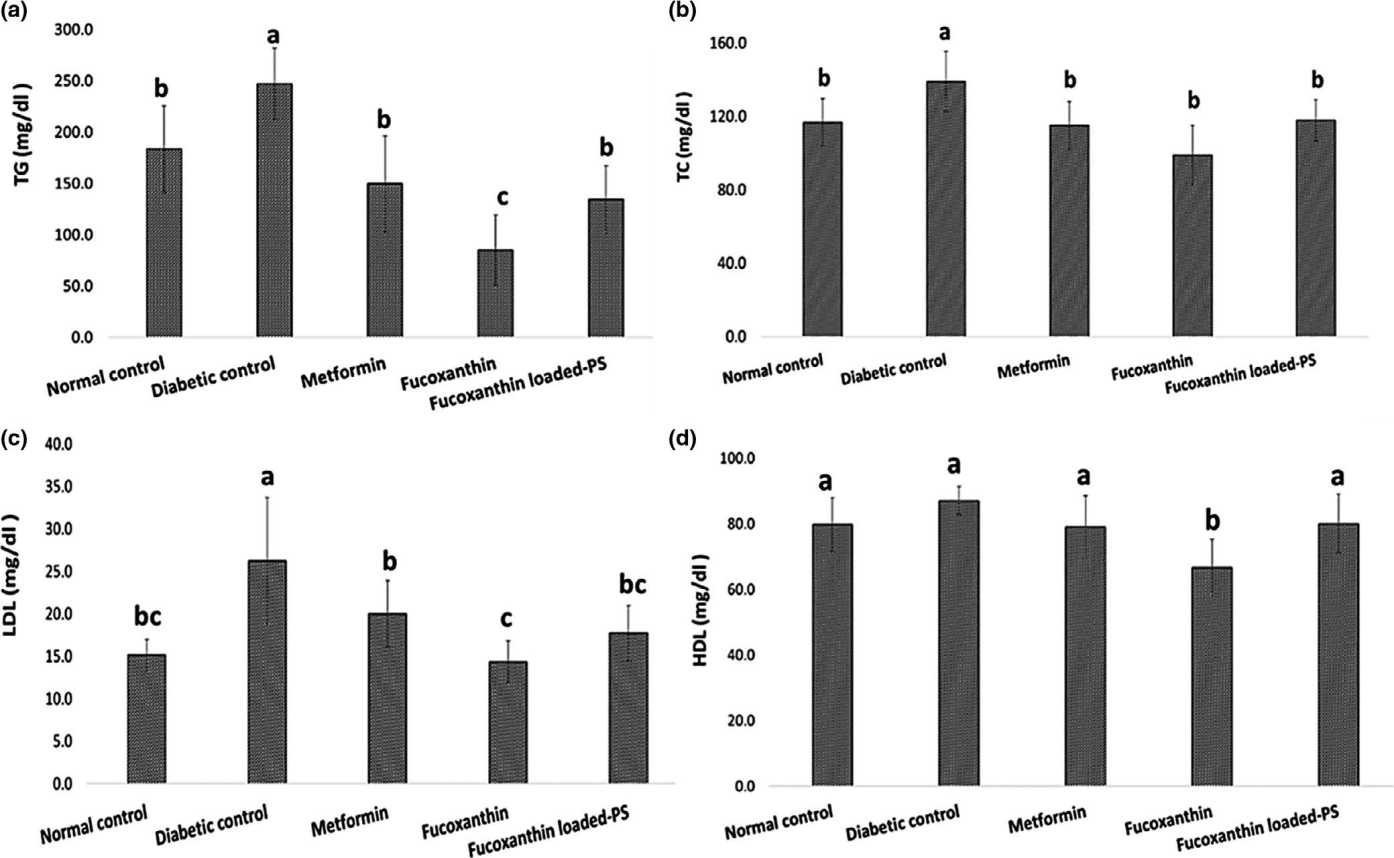
Lipid profile of different treatment groups; PS: porous starch, TG: triglycerides, TC: total cholesterol, HDL: high‐density lipid, LDL: low‐density lipid. Data are expressed as mean ± *SD* (*n* = 3). Different letters indicate significant differences (*p* < .05)

The lipid profile investigation (Figure 3) revealed that the diabetic group in all parameters of the serum lipid profile, except for HDL, were significantly higher (*p* < .05) than other groups. Compared to positive control (metformin), the treated group with fucoxanthin showed the significantly reduced levels of TG, LDL, and HDL (Figure 3). Fucoxanthin‐loaded PS, and metformin‐treated groups had the same effect on all parameters of lipid profile and significantly reduced the levels of TG, TC, and LDL (*p* < .05). The main antiobesity mechanism of fucoxanthin is inhibiting intercellular lipid accumulation through UCP‐1 expression in WAT (Maeda et al., 2009). Moreover, the interaction between macrophages and adipocytes causes overexpression of pro‐inflammatory products in obese WAT, inhibiting the macrophage infiltration and consequently suppressing TNF‐α overexpression (Hosokawa et al., 2010). There was also a significant decrease in TG and TC after administration of 0.4% w/w fucoxanthin for 6 weeks in C57BL/Ksj‐db/db. Expression of several genes, including peroxisome proliferation‐activated receptor alpha, p‐acetyl CoA carboxylase, carnitine palmitoyl transferase 1, has been reported following the fucoxanthin administration. These are key proteins in regulating fatty acids synthesis and oxidation (Y. Zhang et al., 2018). Several studies have confirmed management of the lipid profile by fucoxanthin administration. For instance, Park et al., (2011) showed that 0.02% fucoxanthin in diet decreased the hepatic lipid droplet accumulation through stimulation of the β‐oxidation activity and inhibition of the phosphatidate phosphohydrolase activity. Phosphatidate phosphohydrolase is the main enzyme responsible for conversion of phosphatidate to diglyceride, precursor of the triglyceride, phosphatidylcholine, and phosphatidylethanolamine (Park et al., 2011). S.‐I. Kang et al., (2012) also reported a reduced triglyceride level in mice fed with *petalonia binghamiae* extract (150 mg kg^‐1^ day^‐1^) for 70 days. They suggested that fucoxanthin administration by increasing LKB1 phosphorylation in mature 3T3L1 adipocytes causes improved AMP‐activated protein kinase (AMPK) activity which influences on β‐oxidation of fatty acids (S.‐I. Kang et al., 2012).

Besides, oxidizing agents such as reactive oxygen species (ROS) play an important role in lipid and protein oxidation, which can be regarded as a predisposing factor toward diabetes (Sharma et al., 2010). Thus, bioactive compounds with antioxidant and anti‐inflammatory activity can reduce the TG level by scavenging the free radicals (Akbarzadeh et al., 2018). In this regard, fucoxanthinol causes downregulation of iNOS and COX‐2 mRNA expression in RAW264.7 macrophage‐like cells, consequently inhibits the overexpression of iNOS in the WAT of obese mice and adipocytes (Hosokawa et al., 2010). Also, the brown algae extracts have the potential to inhibit oxidative stress and the lipid accumulation in liver (Song et al., 2018).

### 3.4 Histological and morphometric analysis of pancreatic tissue

**FIGURE 4:**
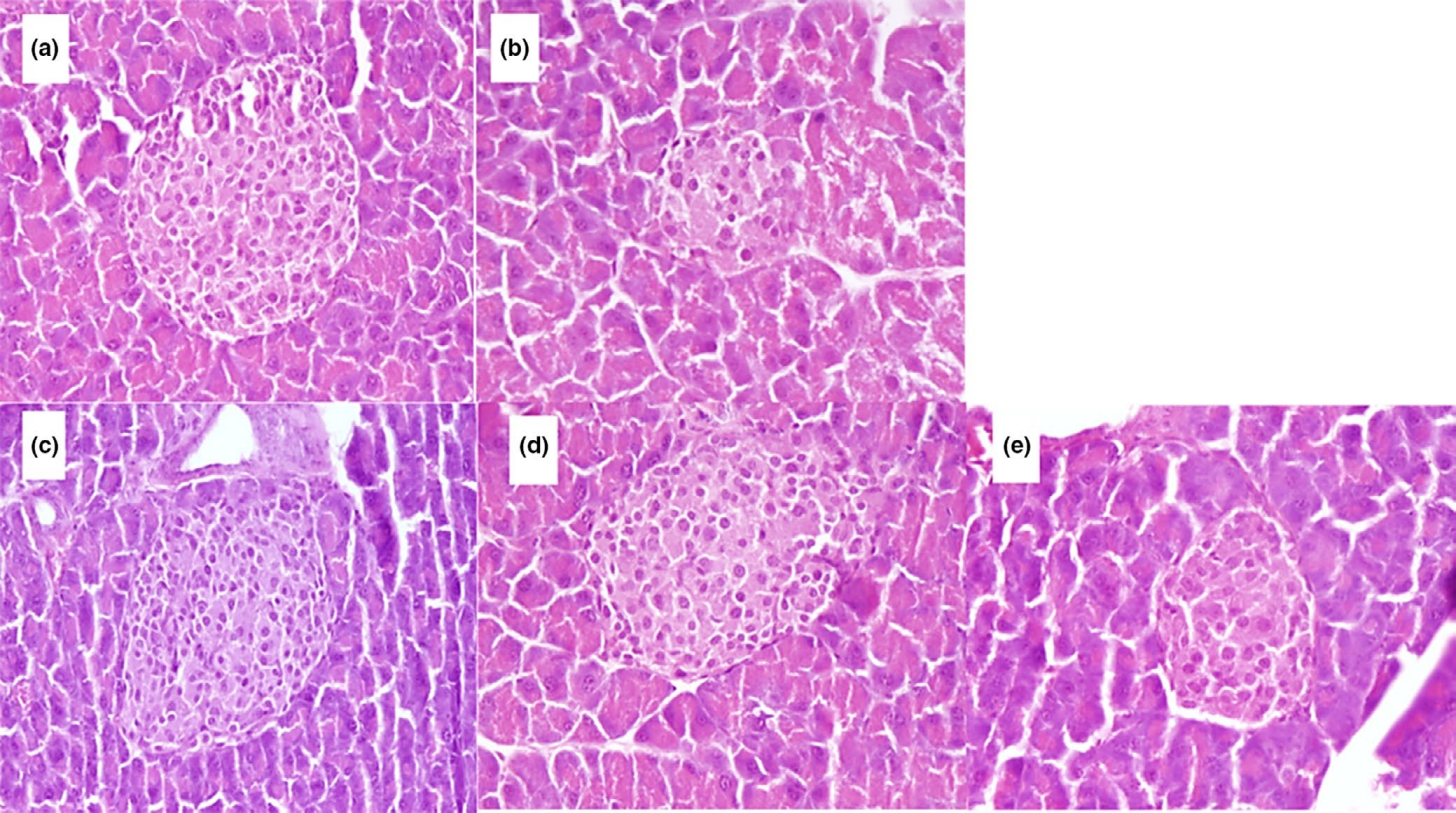
The histopathology of pancreatic tissue in (a) normal control, (b) diabetic control, (c) metformin, (d) fucoxanthin, and (e) fucoxanthin‐encapsulated PS‐treated mice groups

The histopathological evaluation of pancreatic tissues is shown in Figure 4. Pancreatic tissue section of nondiabetic mice showed the normal appearance of islets of Langerhans (Figure 4a). According to Figure 4b, the STZ caused marked degenerative changes of islet cells of diabetic mice with a reduction in size and number, while pancreatic beta cells in diabetic groups were recovered and proliferated after treatment with metformin or fucoxanthin.

Indeed, STZ has a selective cytotoxic action on the beta cells and can generate free radicals which destruct beta cells DNA chains and consequently result in dysregulation of the pancreatic beta cells functions, such as impaired glucose oxidation, reduction of insulin synthesis and secretion and disruption of glucose transport and glucokinase activity (Ghasemi et al., 2014; Koneri et al., 2018).

Sections prepared from the pancreas of the treatment groups showed tissue regeneration, specially mice group fed with metformin at 50 mg/kg (Figure 4c). Indeed, the diabetic group treated with metformin exhibited the area of pancreatic islets similar to the nondiabetic control mice. Metformin exhibited antidiabetic activity by reducing insulin resistance through reduced hepatic glucose production, increased glucose consumption by muscles, or decreased intestinal glucose absorption (Erejuwa, 2014).

Like metformin‐fed mice, the *Sargassum* extract‐rich of fucoxanthin showed the regenerative effect (Figure 4d). Also, the protecting effect of fucoxanthin on shrinkage of cells was similar to metformin. On the other hand, the roundness of the pancreatic islets did not show any significant differences between the free fucoxanthin and the metformin‐treated groups. It seems that fucoxanthin may diminish the oxidative stress caused by hyperglycemia in pancreatic beta cells. However, fucoxanthin‐encapsulated PS had lower influence on the pancreas tissue possibly because of the slow release of encapsulated fucoxanthin as reported in our previous study (Najme Oliyaei et al., 2020).

Generally, hyperglycemia, inflammatory cytokines, hyperlipidemia, or oxidative stress can result in dysfunction of pancreatic beta cell. The low levels of antioxidant enzyme expressions in the beta cells make them liable to oxidative stress by reactive oxygen and nitrogen species (Batumalaie et al., 2014). Thus, any component with antioxidant activity such as *Sargassum* extract‐rich fucoxanthin may have the regenerative effect.

Similar observation was reported by Akbarzadeh et al., (2018) who evaluated the antidiabetic effect of *Sargassum oligocystum* on the STZ‐induced rat. The hydroalcoholic extract at 300 mg/kg dose after 30‐day treatment regenerated the beta cells, which was attributed to the high polyphenols’ contents and antioxidant activity of the extract. Moreover, Koneri et al., (2018) revealed the proliferating activity of algae extract on islet cells of type I diabetic rats. The histological evaluations of STZ‐induced pancreatic damage in rats exhibited significant reduction in beta cell density, whereas the methanolic extracts of *Sargassum polycystum* and *Gracilaria edulis* caused increased beta cell density in the treated diabetic rats, indicating insulin secretagogue activity. Murugesan et al., (2016) also reported that oral administration of the methanolic extracts of *S*. *fusiformis* and *P*. *hornemannii* (10 mg/kg) caused regenerative changes in tissue architecture of Islet cells of pancreas and revealed higher persistence against necrotic changes rather than alloxan‐induced diabetic rat.

## 4 CONCLUSIONS

The present study confirmed the appreciable antidiabetic effect of fucoxanthin in the STZ‐induced diabetic mice model. Decreased blood glucose was obtained by administration of fucoxanthin. Although blood glucose‐lowering effect of the encapsulated fucoxanthin in PS was less than free fucoxanthin, the dose of 400 mg/kg exhibited therapeutic efficacy in type 2 diabetic mice model. Fucoxanthin can reduce also the lipid profile parameters such as TG, TC, LDL, and HDL. Moreover, in the diabetic mice, treatment with fucoxanthin or fucoxanthin‐encapsulated PS as well as metformin led to regeneration of pancreatic beta cells. This study confirms the antidiabetic effect of fucoxanthin after oral administration in the STZ‐induced diabetic mice. In addition, the results clearly indicate that the effect of fucoxanthin on the glucose metabolism can last for a prolonged period in our experimental animals.

## CONFLICT OF INTEREST

The authors declare no conflict of interest.

## AUTHOR CONTRIBUTION

**Najme Oliyaei:** Conceptualization (equal); Data curation (equal); Formal analysis (equal); Investigation (equal); Methodology (equal); Writing‐original draft (equal). **Marzieh Moosavi‐Nasab:** Conceptualization (equal); Funding acquisition (equal); Project administration (equal); Resources (equal); Supervision (equal); Validation (equal); Writing‐review & editing (equal). **Ali Mohammad Tamaddon:** Conceptualization (equal); Methodology (equal); Project administration (equal); Validation (equal); Writing‐review & editing (equal). **Nader tanideh:** Methodology (equal); Project administration (equal); Resources (equal); Validation (equal); Writing‐review & editing (equal).

## Data Availability

Research data are not shared.
